# Transient left ventricular dysfunction following chimeric antigen receptor T‐cell‐mediated encephalopathy: A form of stress cardiomyopathy

**DOI:** 10.1002/jha2.369

**Published:** 2021-12-14

**Authors:** Adam Khorasanchi, Amir M. Ansari, Wendy Bottinor, Gary Simmons, Antonio Abbate, Amir A. Toor

**Affiliations:** ^1^ Department of Internal Medicine Massey Cancer Center, Virginia Commonwealth University Richmond Virginia USA; ^2^ Division of Cardiology Virginia Commonwealth University Richmond Virginia USA

**Keywords:** cardiovascular toxicity, chimeric antigen receptor T‐cell therapy, cytokine release syndrome, diffuse large B‐cell lymphoma, encephalopathy, stress cardiomyopathy

## Abstract

Chimeric antigen receptor (CAR) T‐cell therapy represents a new strategy in treating lymphoid malignancies, such as relapsed‐refractory diffuse large B‐cell lymphoma (DLBCL). Several toxicities including cytokine release syndrome (CRS), neurotoxicity, and cardiovascular toxicity have been linked to CAR T‐cell therapy. Transient impairment in left ventricular systolic function is described after CAR‐T, however, the mechanism remains poorly understood. This paper reports the clinical presentation and outcome of two patients with relapsed‐refractory DLBCL who experienced encephalopathy and CRS following CAR T‐cell therapy and developed transient left ventricular dysfunction consistent with stress cardiomyopathy.

## 1 INTRODUCTION

Chimeric antigen receptor T (CAR‐T) cells have emerged as a promising therapy for patients with refractory adult hematologic malignancies. Cardiovascular (CV) toxicity, such as transient left ventricular (LV) systolic dysfunction has been observed following CAR‐T cell therapy, however the mechanism remains poorly understood [1, 2]. This paper presents two adult patients with diffuse large B‐cell lymphoma (DLBCL) who experienced encephalopathy and CRS following CAR‐T and developed stress cardiomyopathy.

## 2 CASE 1

**FIGURE 1:**
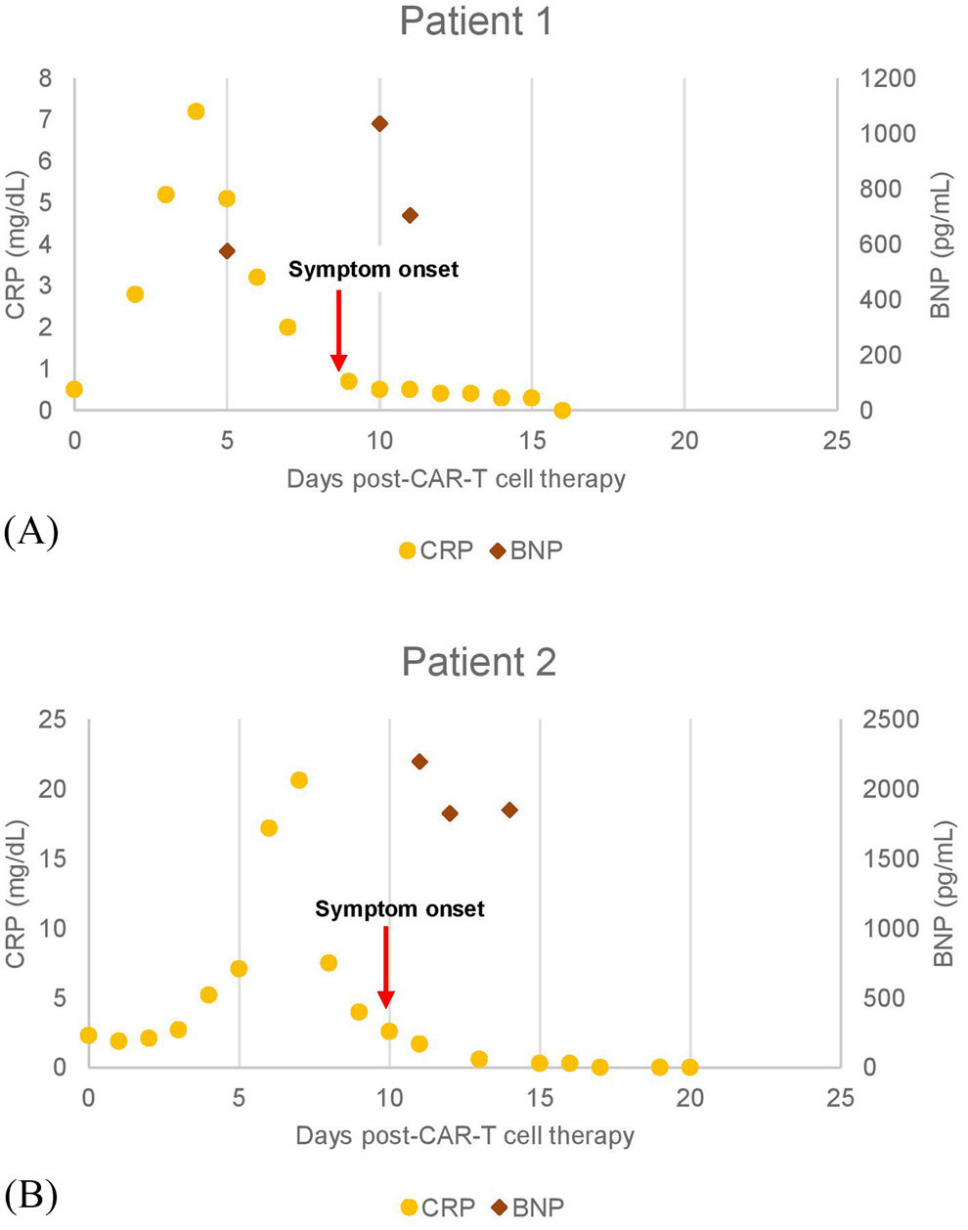
C‐reactive protein (CRP, left *Y*‐axis) and brain natriuretic peptide BNP, right *Y*‐axis) levels following CAR‐T‐cell therapy measured in days (*X*‐axis). Baseline CRP levels for patient 1 (A) and patient 2 (B) prior to CAR‐T were 2.3 and 0.5 mg/dl, respectively. BNP levels prior to CAR‐T for A‐B were not available. The red arrow indicates the day of CV symptom onset. CRP levels for A‐B peaked at approximately day +5 and day +7, respectively. BNP levels following CAR‐T were elevated in A‐B secondary to acute systolic LV dysfunction. Symptom onset (red arrow) was noted on day +8, and day +10, respectively. CRP and BNP levels in A‐B decreased following symptom resolution

A 76‐year‐old female presented with DLBCL, refractory to two prior therapies. CV history was significant for hypertension, hyperlipidemia, deep vein thrombosis, and transient ischemic attack. Left ventricular ejection fraction (LVEF) 2 months pre‐CAR‐T was 54%. Fludarabine 30 mg/m^2^/day and cyclophosphamide 300 mg/m^2^/day given for 3 days (Flu/Cy) was followed by infusion of 2.0 × 10^8^ CAR‐T cells/kg (axicabtagene ciloleucel, Yescarta, Kite Pharma, USA). The hospital course was complicated by grade 1 and grade 3 CRS on day +1 and day +6, respectively [3]. The patient was treated supportively, and four tocilizumab doses of 8 mg/kg were administered, given 18 h apart. On day +4, atrial fibrillation with rapid ventricular response developed, and was treated with intravenous metoprolol and oral amiodarone. On day +9, the patient experienced lethargy, concerning for immune effector cell‐associated neurotoxicity (ICANS). Magnetic resonance imaging (MRI) brain showed acute/subacute ischemic infarcts of the right frontal periventricular matter and left posterior frontal lobe. These symptoms resolved 3 days later following supportive care. During this period of encephalopathy, severe bilateral lower extremity pain associated with cyanosis of the feet developed. Laboratory evaluation demonstrated lactate 2.3 mmol/L, brain natriuretic peptide (BNP) 1037 pg/ml, and troponin‐I (Tn‐I) 0.25 ng/ml (Figure 1). Electrocardiogram showed sinus rhythm, nonspecific ST/T changes, and QT prolongation. Echocardiogram demonstrated acute systolic LV dysfunction, LVEF of 30%, global apical akinesis, and relative preservation of basal function with apical ballooning. Intravenous heparin was given for microvascular ischemia, with significant improvement of pain and cyanosis. Echocardiogram on day +15 showed improved LV systolic function, LVEF of 50%, consistent with stress cardiomyopathy. The patient was discharged on day +21, and has remained symptom free 12 months later.

## 3 CASE 2

**FIGURE 2:**
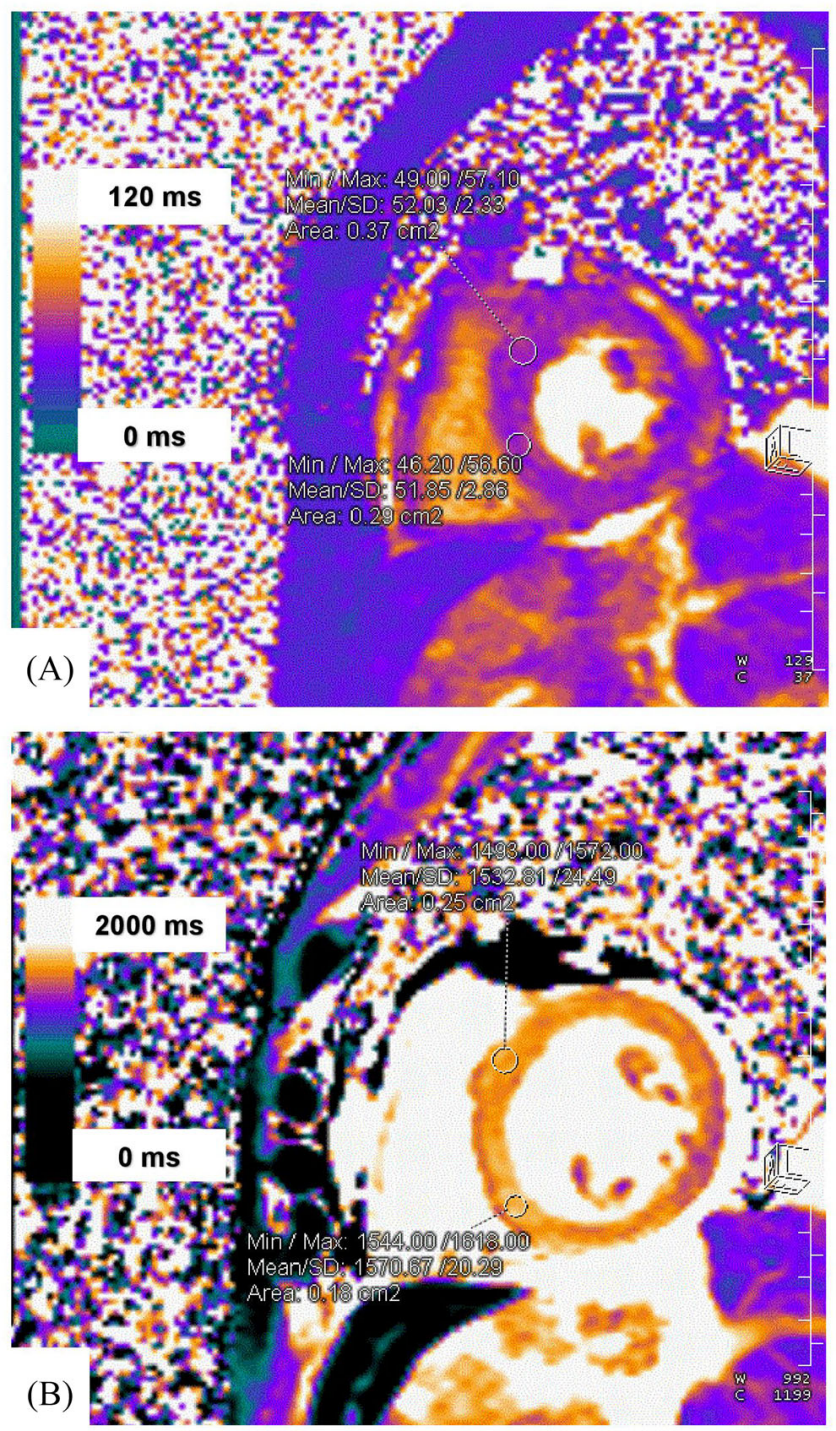
Cardiac MRI native T1 and T2 maps with color scale (obtained on a Siemens Vida 3‐Tesla MRI scanner) of patient 2 is shown above. Native T2 values (A) were increased throughout the myocardium with several values greater than 50 ms (upper limit normal 38 ± 3.5 ms). Native T1 values (B) were also diffusely increased with several values greater than 1550 ms (upper limit normal 1227 ms). The increased T1 and T2 values were most prominent in the segments with regional wall motion abnormalities (basal and mid segments of the septum and anterior walls), and no delayed enhancement was appreciated following IV contrast. These findings were most consistent with the diagnosis of stress cardiomyopathy

A 61‐year‐old female presented with DLBCL, refractory to two prior therapies. There was no previous CV history, and LVEF 1 month pre‐CAR‐T was 55%. Following Flu/Cy immunoablation, axicabtagene ciloleucel was administered at 2.0 × 10^6^ CAR‐T cells/kg. Grade 2 CRS developed on day +6, which was managed supportively, and a single tocilizumab dose 8 mg/kg.^3^ On day +7, encephalopathy developed grade 4 ICANS [3]. High‐dose methylprednisolone (MP) 1 g/day and a second tocilizumab dose were administered. Mental status gradually improved following a 3‐day course of MP 1 g/day. The MP was tapered to 250 mg twice daily on day +10. Shortly following MP dose reduction, the patient became acutely dyspneic with worsening hypoxia. Electrocardiogram showed new *T* wave inversion in anteroseptal leads and QT prolongation. CT angiogram of the chest revealed pleural effusions and global cardiomegaly. Laboratory studies demonstrated lactate 3.2 mmol/L, BNP 2198 pg/ml, and Tn‐I 0.14 ng/ml (Figure 1). Echocardiogram showed reduced LVEF of 25%, akinesis of the mid‐ventricular sections, relative sparing of the apex, and hypokinesis of the bases in a noncoronary distribution. A third tocilizumab dose was given, along with supportive care, with significant symptom improvement. Cardiac MRI (CMR) on day +16 revealed reduced LV systolic function, LVEF of 35%, and regional LV hypokinesis in the mid segments consistent with mid‐ventricular stress cardiomyopathy. No delayed enhancement was appreciated following gadolinium‐based contrast administration. Native T1 and T2 values were increased throughout the myocardium, most prominent in segments with regional motion abnormalities [4] (Figure 2). The patient experienced no further complications and was discharged on day +23 to complete a prednisone taper. Day +40 echocardiogram demonstrated resolution of systolic dysfunction, LVEF of 55%, confirming stress cardiomyopathy.

## 4 DISCUSSION

CAR‐T associated CV toxicity is a serious therapy‐related complication, the understanding of which is hampered by a paucity of data from clinical trials [5]. The true incidence is unknown, however studies in adult patients have reported an incidence of 12%–21% [6, 7]. Similar to our cases, the median onset duration of CV toxicity following CAR‐T infusion is 12.5 days (range 2–24 days) [8]. A wide spectrum of clinical manifestations exists, including dysrhythmias and LV systolic dysfunction [2]. LV systolic dysfunction has been observed in patients with elevated troponin and those with grade 2 CRS or greater [6].

While most cases of CAR‐T associated CV toxicity occur secondary to high‐grade CRS [3, 9], it can also happen in patients who are not in shock and in some may be associated with encephalopathy. Interestingly, neurotoxicity has been shown to be closely linked with CV toxicity and CRS [10]. The pathophysiology for this form may be similar to stress cardiomyopathy [11]. Stress cardiomyopathy is defined as acute and transient LV systolic dysfunction mediated by a neuro‐cardiogenic mechanism and classically precipitated by an emotionally or physically stressful event. Both of our patients’ cardiotoxicity were preceded by physical stressors ICANS and CRS, resulting in cardiomyopathy. The presence of reversible LV regional wall motion abnormalities extending beyond a single epicardial coronary artery distribution is consistent with this diagnosis [4]. During the acute phase of stress cardiomyopathy, T_2_ weighted CMR may show myocardial edema as high signal intensity in the acute phase. Lack of delayed gadolinium enhancement on CMR is also suggestive of this diagnosis, as seen with our second patient [4].

Management of CAR‐T associated CV toxicity includes supportive measures and the use of tocilizumab, an IL‐6 receptor antagonist [9]. Further studies are needed to define the role of corticosteroids for the treatment of CV events [12]. The prognosis of these patients is not well understood [2]. Cardiomyopathy is reversible in some, as demonstrated in a recent study, in which LVEF recovered in 75% of patients with supportive care [8, 11]. However, CAR‐T cells can persist more than 10 years posttreatment, and therefore potential long‐term CV effects remain unclear [11].

In conclusion, this paper supports the potential neuro‐cardiogenic mechanism of CAR‐T associated CV toxicity as both of our patients developed stress cardiomyopathy with encephalopathy and CRS as the likely triggers. However, given the small sample size of our case series, further studies are needed to confirm these findings.

## CONFLICT OF INTEREST

The authors declare no conflict of interest.

## AUTHOR CONTRIBUTIONS

All authors participated in the writing of this manuscript and give final approval of the version submitted.

## References

[1] Ghosh AK , Chen DH , Guha A , Mackenzie S , Walker JM , Roddie C , et al. CAR T‐cell therapy‐related cardiovascular outcomes and management: systemic disease or direct cardiotoxicity? J Am Coll Cardiol CardioOnc. 2020;2:97–109. 10.1016/j.jaccao.2020.02.011. PMC8352125 34396213

[2] Dal'bo N , Patel R , Parikh R , Shah SP , Guha A , Dani SS , et al. Cardiotoxicity of contemporary anticancer immunotherapy. Curr Treat Options Cardiovasc Med 2020;22:62. 10.1007/s11936-020-00867-1. 33162729 PMC7605901

[3] Lee DW , Santomasso BD , Locke FL , Ghobadi A , Turtle CJ , Brudno JN , et al. ASTCT consensus grading for cytokine release syndrome and neurologic toxicity associated with immune effector cells. Biol Blood Marrow Transplant. 2019;25:625–38. 10.1016/j.bbmt.2018.12.758. 30592986 PMC12180426

[4] Medina de Chazal H , Del Buono MG , Keyser‐Marcus L , Ma L , Moeller FG , Berrocal D , et al. Stress cardiomyopathy diagnosis and treatment: JACC state‐of‐the‐art review. J Am Coll Cardiol. 2018;72:1955–71. 10.1016/j.jacc.2018.07.072. 30309474 PMC7058348

[5] Catino AB . Cytokines are at the heart of it: cytokine release syndrome underlies cardiovascular effects of CAR T‐cell. J Am Coll Cardiol CardioOnc. 2020;2:204–6. 10.1016/j.jaccao.2020.05.006. PMC8352284 34396229

[6] Alvi RM , Frigault MJ , Fradley MG , Jain MD , Mahmood SS , Awadalla M , et al. Cardiovascular events among adults treated with chimeric antigen receptor T‐cells (CAR‐T). J Am Coll Cardiol. 2019;74:3099–108. 10.1016/j.jacc.2019.10.038. 31856966 PMC6938409

[7] Lefebvre B , Kang Y , Smith AM , Frey NV , Carver JR , Scherrer‐Crosbie M . Cardiovascular effects of CAR T cell therapy. A retrospective study. JACC CardioOncol. 2020;2:193–203. 10.1016/j.jaccao.2020.04.012. 32776016 PMC7413146

[8] Ganatra S , Redd R , Hayek SS , Parikh R , Azam T , Yanik G , et al. Chimeric antigen receptor T‐cell therapy‐associated cardiomyopathy in patients with refractory or relapsed non‐hodgkin lymphoma. Circulation 2020;142:1687–90. 10.1161/CIRCULATIONAHA.120.048100. 33104402

[9] Jamal FA , Khaled SK . The cardiovascular complications of chimeric antigen receptor T cell therapy. Curr Hematol Malig Rep. 2020;15:130–2. 10.1007/s11899-020-00567-4. 32016789

[10] Guha A , Addison D , Jain P , Gutierrez JM , Ghosh A , Roddie C , et al. Cardiovascular events associated with chimeric antigen receptor T cell therapy: cross‐sectional FDA adverse events reporting system analysis. Biology of blood and marrow transplantation: J Am Soc Blood Marrow Transplant. 2020;26:2211–16. 10.1016/j.bbmt.2020.08.036. 32966880

[11] Asnani A . Cardiotoxicity of immunotherapy: incidence, diagnosis, and management. Curr Oncol Rep. 2018;20:44. 10.1007/s11912-018-0690-1. 29644505

[12] Burns EA , Gentille C , Trachtenberg B , Pingali SR , Anand K . Cardiotoxicity associated with anti‐CD19 chimeric antigen receptor T‐cell (CAR‐T) therapy: recognition, risk factors, and management. Diseases 2021;9:20. 10.3390/diseases9010020. 33802788 PMC8006027

